# Nonlinear Association Between THs, TSH, and HbA1c in Patients With Type 2 Diabetes Mellitus: A Retrospective Study

**DOI:** 10.1111/1753-0407.70200

**Published:** 2026-03-09

**Authors:** Mengjie Chen, Yuqin Gan, Fengxiang Tian, Yun Bao, Zhonglan Chen

**Affiliations:** ^1^ Department of Cardiology West China Hospital, Sichuan University Chengdu Sichuan China; ^2^ West China School of Nursing Sichuan University Chengdu Sichuan China; ^3^ West China School of Public Health and West China Fourth Hospital Sichuan University Chengdu Sichuan China; ^4^ School of Nursing Chengdu Medical College Chengdu Sichuan China

**Keywords:** glycated hemoglobin, nonlinear association, thyroid hormone, thyroid stimulating hormone, type 2 diabetes mellitus

## Abstract

**Aims:**

This study aimed to investigate the potential non‐linear relationships between thyroid hormones and thyroid‐stimulating hormone levels on glycemic control levels.

**Methods:**

A retrospective analysis of electronic medical records was performed on patients with T2DM who received treatment at a tertiary care hospital in Chengdu, Sichuan Province, between 2018 and 2023. RCS regression and threshold effect analyses were employed to assess potential nonlinear associations among THs, TSH, and glycemic control.

**Results:**

Data from a total of 1413 patients were included in the analysis. RCS regression revealed a significant non‐linear association between FT3 and HbA1c (*p* for nonlinearity < 0.05). Threshold effect analysis demonstrated no statistically significant inflection point for FT3 in relation to HbA1c (FT3 < 5.92 pmol/L, *β* = 0.008, 95% CI (−0.122, 0.281); FT3 > 5.92 pmol/L, *β* = −0.109, 95% CI (−0.261, 0.042)). FT4 exhibited a significant non‐linear relationship with HbA1c (*p* for nonlinearity < 0.05), identifying an inflection point at 14.82 pmol/L. Below this threshold, each 1 pmol/L increase in FT4 was associated with a 0.263% elevation in HbA1c (*β* = 0.263, 95% CI 0.189–0.337). Similarly, TSH demonstrated a non‐linear association with HbA1c (*p* for nonlinearity < 0.05), with an inflection point identified at 5.53 mIU/L. When TSH was below 5.53 mIU/L, each 1 mIU/L increase was associated with a 0.179% reduction of HbA1c (*β* = −0.179, 95% CI −0.281 to −0.076).

**Conclusion:**

Nonlinear associations were observed between thyroid function markers (FT3, FT4, and TSH) and HbA1c levels in patients with T2DM. These findings provide novel evidence for understanding thyroid‐glucose metabolic interactions.

## Highlights

- We used restricted cubic spline modeling and combined it with threshold effects analysis to explore the non‐linear relationship between thyroid hormone, thyroid‐stimulating hormone, and blood glucose levels in patients with T2DM.
- Non‐linear associations between FT3, FT4, TSH, and glycated hemoglobin in patients with T2DM.

## Graphical abstract

Non‐linear Association: FT4 and TSH display significant non‐linear relationships with HbA1c in T2DM patients. FT4 Threshold (14.82 pmol/L): Below this inflection point, FT4 is positively associated with HbA1c. TSH Threshold (5.53 mIU/L): Below this inflection point, TSH is inversely associated with HbA1c. Provides novel evidence regarding the interaction between thyroid function and glucose metabolism.

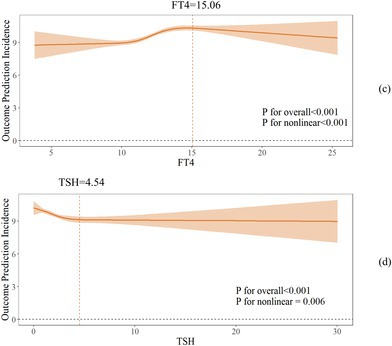

## 1 Introduction

Diabetes mellitus poses a major public health challenge, with its global incidence rising steadily in recent decades. Type 2 diabetes mellitus (T2DM) represents the predominant form of diabetes, responsible for over 90% of global diabetes cases [1]. The overall prevalence of T2DM in China has reached 14.92%, and the number of diabetic patients has exceeded 140 million, ranking first globally [2]. This epidemic imposes considerable economic burdens and health risks at both societal and familial levels [1].

Hyperglycemia is a hallmark of diabetic metabolic abnormalities, primarily resulting from pancreatic β‐cell dysfunction [3, 4]. Chronic hyperglycaemia is associated with microvascular and macrovascular complications, reduced quality of life, and premature mortality [5]. Glycated hemoglobin (HbA1c) serves as a critical indicator for diabetes management, reflecting long‐term glycemic control over the preceding 2–3 months [6]. Elevated HbA1c levels are strongly associated with an increased risk of diabetes‐related complications and mortality [7]. Proactive management of HbA1c in individuals with diabetes is essential for attenuating these risks.

Thyroid hormones (THs), which are critical endocrine regulators in humans, play a pivotal role in regulating glucose metabolism [8, 9]. They modulate glucose homeostasis through pancreatic β‐cell activity and coordinated metabolic interactions among multiple organs, including the liver, pancreas, etc. [10]. Additionally, THs antagonize insulin's physiological effects, suppressing hepatic glucose production and impairing muscular glucose uptake [11]. Concurrently, the body's Thyroid‐stimulating hormone (TSH) levels are closely associated with neurocognitive deficits, obesity, dyslipidemia, T2DM, and related comorbidities [12]. Thyroid dysfunction can induce insulin resistance, cause blood glucose fluctuation, and aggravate glucose metabolism disorders. This not only increases the incidence and severity of diabetes but also complicates its clinical management [13, 14, 15, 16].

Rising blood glucose indicates that marked insulin resistance, which may be attributable to TSH and THs; this condition requires early intervention to achieve stricter glycemic control blood and improve the prognosis [17]. However, the precise regulatory effects of TH and TSH on blood glucose homeostasis remain debated. Some studies have indicated a positive correlation between TSH levels and HbA1c [18, 19], while others suggest that elevated TSH enhances insulin sensitivity, potentially leading to hypoglycemia [20]; notably, studies in diabetic populations have further observed that both TSH and Free triiodothyronine (FT3) are negatively correlated with HbA1c [12, 21]. In individuals with T2DM, even subtle variations in thyroid hormone levels may significantly influence blood glucose regulation. Importantly, most previous studies have relied on linear models, which could overlook potential threshold effects or non‐linear relationships.

To address this gap, we conducted a retrospective study to evaluate the nonlinear associations and threshold effects between FT3, FT4, TSH, and HbA1c using restricted cubic spline regression and segmented modeling. The findings from this study may provide new evidence for the mechanism of thyroid‐glucose metabolism interactions in T2DM, offering a novel theoretical basis and revealing potential therapeutic targets to improve glycemic control and clinical outcomes in patients with T2DM.

## 2 Methods

### 2.1 Design and Participants

This retrospective cohort study enrolled 1549 patients with T2DM from the Standardized Metabolic Management Center (MMC) at the Department of Endocrinology, a tertiary care hospital in Chengdu, China, between January 2018 and December 2023.

Inclusion criteria: (1) Diagnosis of T2DM according to the WHO 1999 diagnosis and classification criteria [22]; (2) Age ≥ 18 years at enrollment.

Exclusion criteria: (1) Concomitant severe chronic diseases (e.g., malignancies, hepatic/renal dysfunction, etc.); (2) Incomplete clinical data; (3) Diagnosis of other endocrine disorders (e.g., adrenal diseases, thyroid‐related conditions, and other endocrine disorders affecting thyroid function, etc.).

### 2.2 Data Collection

We collected general demographic and laboratory‐related data from the patients. Demographic variables primarily included age, sex, height, and weight. Body mass index (BMI) was calculated as the ratio of weight to height squared (kg/m^2^). BMI (kg/m^2^) = weight (kg)/height^2^ (m) [23].

The primary laboratory data included three thyroid function tests: free triiodothyronine (FT3), free tetraiodothyronine (FT4), and thyroid stimulating hormone (TSH). The reference ranges for these thyroid function parameters were as follows: FT3, 3.53–7.37 pmol/L; FT4, 7.98–16.02 pmol/L; and TSH, 0.56–5.91 mIU/L, based on the reference standards of the hospital's Laboratory Department.

### 2.3 Statistical Analysis

Data were exported from EpiData (Chinese version) management software and analyzed using IBM SPSS 27.0 and R 4.4. Quantitative data were presented as the mean ± standard deviation ($\bar{\chi }$ ± SD) or median with interquartile range (*M* [*Q*1, *Q*3]), while qualitative data were expressed as *n* (%). Restricted cubic spline (RCS)‐fitted multiple linear regression models were used to assess the nonlinear associations between the three thyroid function parameters and HbA1c, the number of knots set to 4, with adjustments for potential confounders, including sex, age, and BMI. Threshold analysis and segmented fitting were performed for each model. All statistical tests were two‐sided, with *p* < 0.05 considered statistically significant.

### 2.4 Ethical Considerations

The study was approved by the Ethics Committee of First Affiliated Hospital of Chengdu Medical College (approval no. 2022cyfyirb‐BA‐Dec01), and it was carried out in accordance with the Code of Ethics of the World Medical Association (Declaration of Helsinki).

## 3 Results

### 3.1 General Demographic Information

A total of 1549 patients with T2DM were collected in this study. After excluding 136 patients with incomplete data, 1413 participants were included in the final analysis. Among these, 814 were male (57.6%) and 599 were female (42.4%). The mean age of the patients was 56.38 ± 12.98 years, with 881 patients (62.3%) aged 60 years or younger. The average BMI was 24.07 ± 3.41 kg/m^2^, and the mean HbA1c level was 9.63% ± 2.58%. Further details are provided in Table 1.

**TABLE 1:** Sociodemographic characteristics of the study subjects (*N* = 1413).

Variables	*N* (%)/Mean ± SD/*M* (*Q*1, *Q*3)	Variable	*N* (%)/Mean ± SD/*M* (*Q*1, *Q*3)
Gender		FT4 (pmol/L)	12.29 ± 2.25
Man	814 (57.6%)	< 7.98	22 (1.6%)
Woman	599 (42.4%)	7.98–16.02	1323 (93.6%)
Age (years)	56.38 ± 12.98	> 16.02	68 (4.8%)
≤ 60 years	881 (62.3%)	TSH (mIU/L)	1.73 (1.17, 2.71)
> 60 years	532 (37.7%)	< 0.56	65 (4.6%)
BMI (kg/m^2^)	24.07 ± 3.41	0.56–5.91	1298 (91.2%)
FT3 (pmol/L)	5.15 ± 1.22	> 5.91	59 (4.2%)
< 3.53	32 (2.3%)	HbA1c (%)	9.63 ± 2.58
3.53–7.37	1361 (96.3%)	≤ 7	275 (19.5%)
> 7.37	20 (1.4%)	> 7	1138 (80.5%)

*Note:* Reference range: FT3, 3.53–7.37 pmol/L; FT4, 7.98–16.02 pmol/L; and TSH, 0.56–5.91 mIU/L, based on the reference standards of the hospital's Laboratory Department.

Abbreviations: BMI: body mass index; FT3: free triiodothyronine; FT4: free tetraiodothyronine; HbA1c: glycated hemoglobin; M: median; Q1: first percentile of quartiles; Q3: third percentile of quartiles; SD: standard deviation; TSH: thyroid‐stimulating hormone thyrotropin.

### 3.2 Nonlinear Association Between FT3 and HbA1c

**FIGURE 1:**
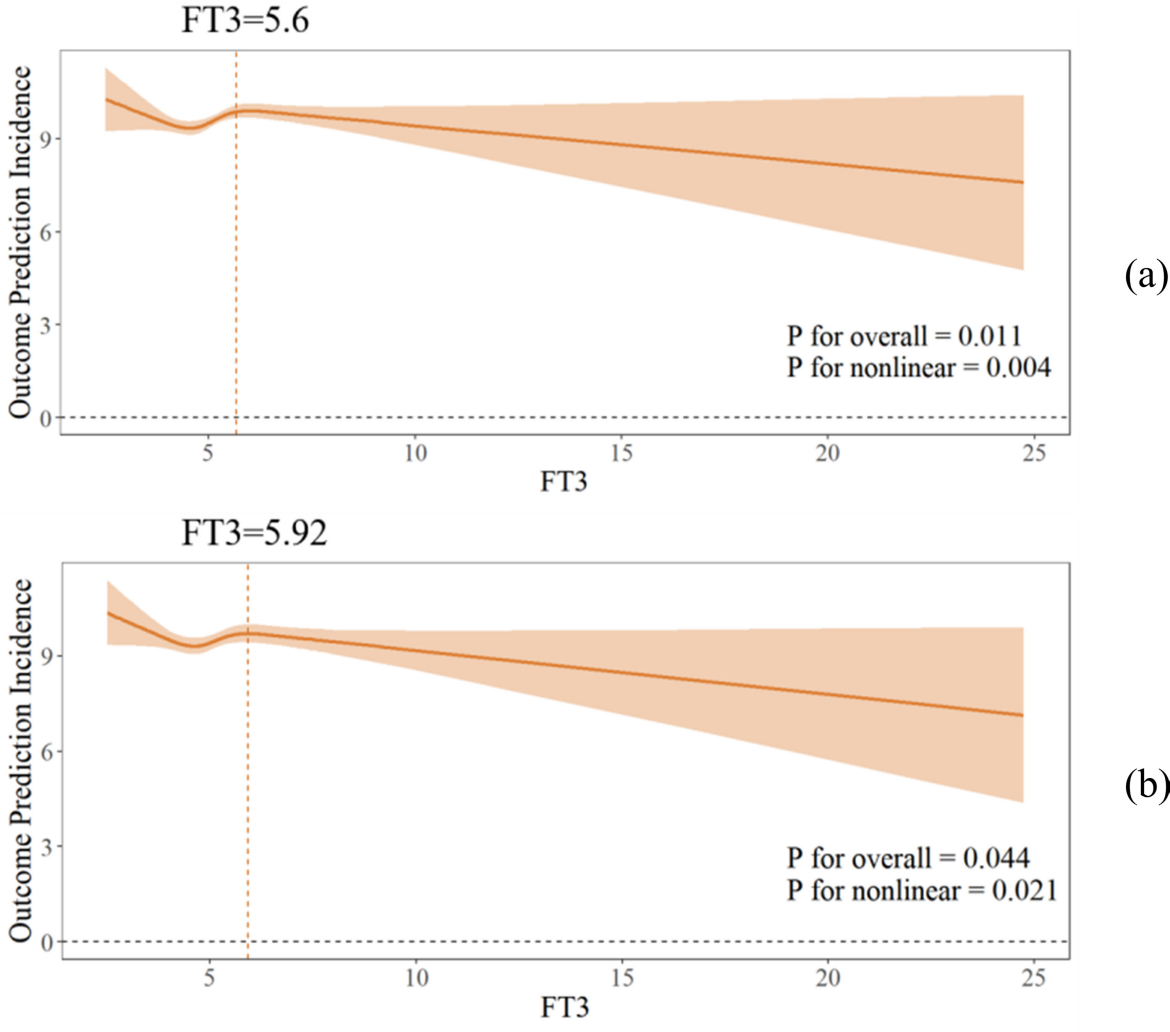
Non‐linear relationship between FT3 and HbA1c: (a) crude model (b) adjusted for age, gender, BMI.

Both the unadjusted model and the model adjusted for confounders exhibited a significant nonlinear association between FT3 and HbA1c (both *p* for nonlinearity < 0.05; Figure 1). In the final model, threshold analysis and segmented regression were performed, revealing an inflection point at FT3 = 5.92 pmol/L. However, no significant association was observed between FT3 and HbA1c when FT3 < 5.92 pmol/L or FT3 > 5.92 pmol/L (FT3 < 5.92: *β* = 0.008, 95% CI (−0.122, −0.281); FT3 > 5.92: *β* = −0.109, 95% CI (−0.261–0.042); *p* for log‐likelihood ratio = 0.176; Figure 1; Table 2). These findings indicate a nonlinear relationship between FT3 and HbA1c, though no distinct threshold effect was identified.

**TABLE 2:** Threshold analysis of the effect of FT3 on HbA1c.

	Crude *β* (95% CI)	Adjusted *β* (95% CI)
Fitting model by standard linear regression	0.019 (−0.092–0.129)	−0.037 (−0.146–0.072)
Fitting model by two‐piecewise linear regression inflection point	5.68	5.92
FT3 < inflection point	0.212 (−0.005, 0.429)	0.008 (−0.122, 0.281)
FT3 > inflection point	−0.086 (−0.237, 0.064)	−0.109 (−0.261, 0.042)
*p* for Log‐likelihood ratio	0.043	0.176

*Note:* Adjusted for age, gender, BMI.

Abbreviations: 95% CI: 95% confidence interval; FT3: Free triiodothyronine; FT4: Free tetraiodothyronine; TSH: thyroid‐stimulating hormone thyrotropin.

### 3.3 Nonlinear Association Between FT4 and HbA1c

**FIGURE 2:**
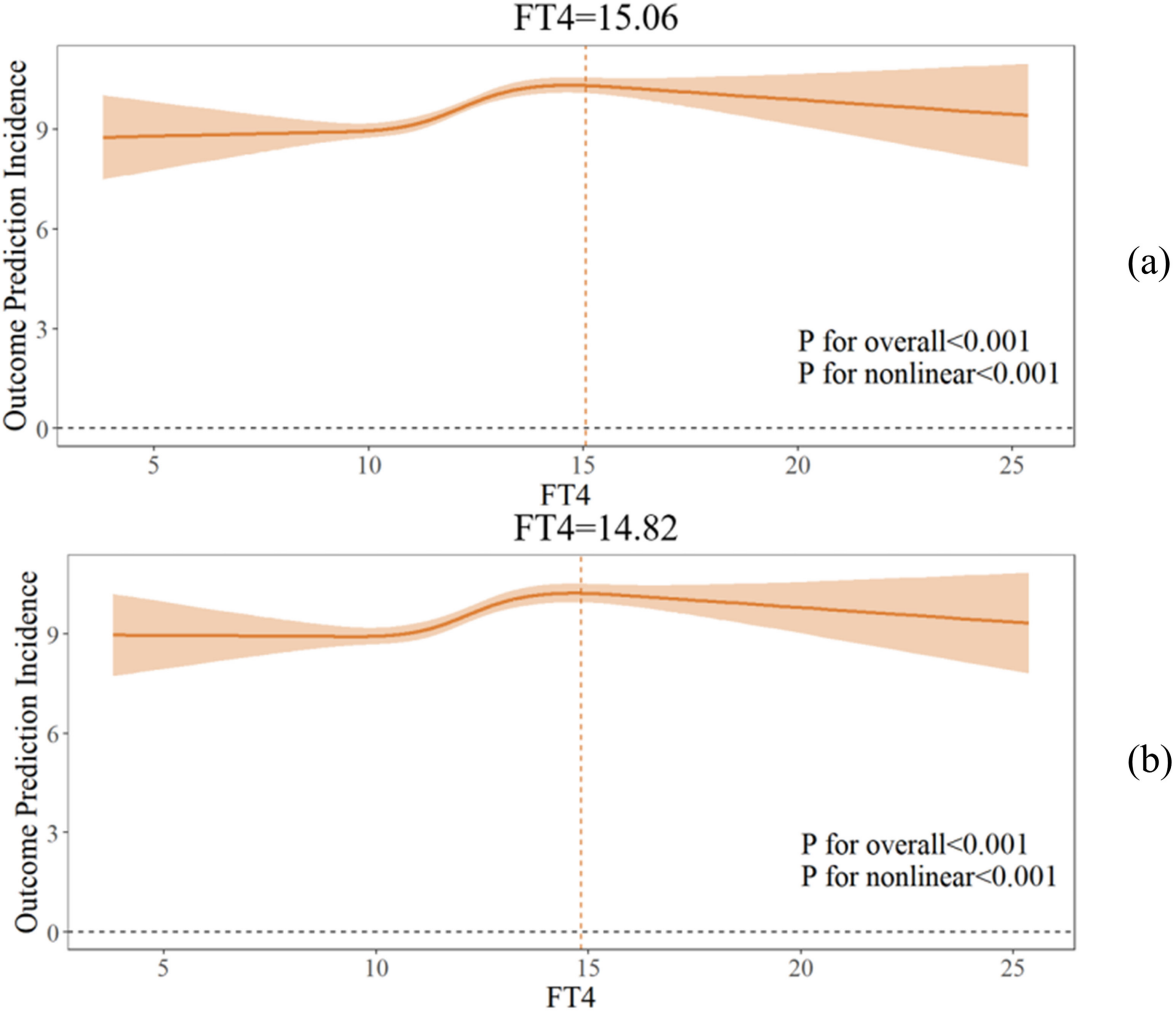
Non‐linear relationship between FT4 and HbA1c: (a) crude model (b) adjusted for age, gender, BMI.

Both the unadjusted model and the model adjusted for confounders exhibited a significant nonlinear association between FT4 and HbA1c (both *p* for nonlinearity < 0.05; Figure 2). In the final model, threshold analysis and segmented regression were performed, revealing an inflection point at FT4 = 14.82 pmol/L. A 0.263% increase in HbA1c was observed per unit increase in FT4 when FT4 < 14.82 pmol/L, but no significant association was found when FT4 > 14.82 pmol/L (FT4 < 14.82: *β* = 0.263, 95% CI (0.189, 0.337); FT4 > 14.82:*β* = −0.116, 95% CI (−0.287, 0.005); *p* for log‐likelihood ratio < 0.001; Figure 2; Table 3).

**TABLE 3:** Threshold analysis of the effect of FT4 on HbA1c.

	Crude *β* (95% CI)	Adjusted *β* (95% CI)
Fitting model by standard linear regression	0.193 (0.134–0.252)	0.179 (0.121–0.236)
Fitting model by two‐piecewise linear regression inflection point	15.06	14.82
FT4 < inflection point	0.278 (0.205, 0.351)	0.263 (0.189, 0.337)
FT4 > inflection point	−0.151 (−0.336, 0.034)	−0.116 (−0.287, 0.055)
*p* for Log‐likelihood ratio	< 0.001	< 0.001

*Note:* Adjusted for age, gender, BMI.

Abbreviations: 95% CI: 95% confidence interval; FT3: free triiodothyronine; FT4: free tetraiodothyronine; TSH: thyroid‐stimulating hormone thyrotropin.

### 3.4 Nonlinear Association Between TSH and HbA1c

**FIGURE 3:**
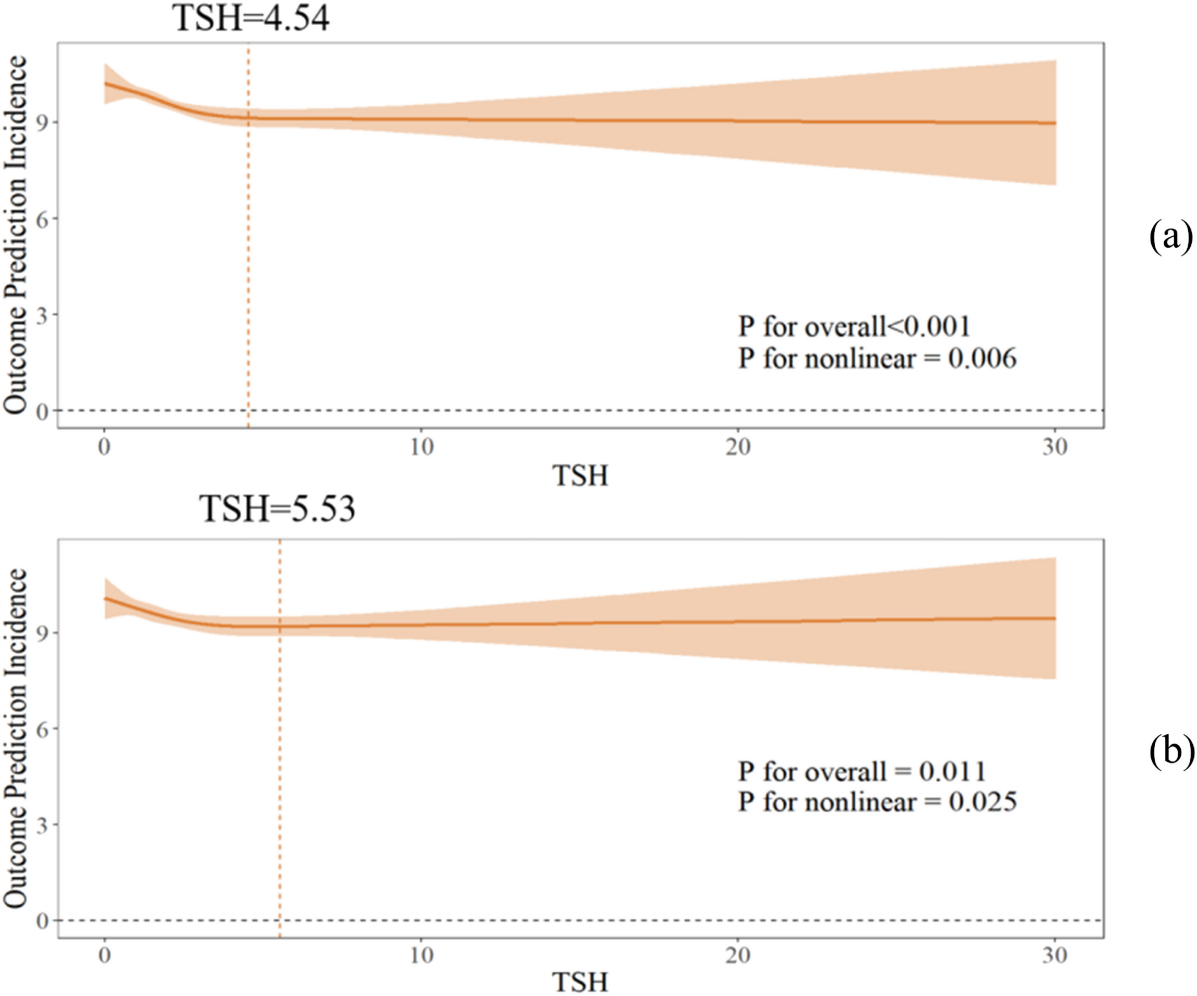
Non‐linear relationship between TSH and HbA1c: (a) crude model (b) adjusted for age, gender, BMI.

Both the unadjusted model and the model adjusted for confounders exhibited a significant nonlinear association between TSH and HbA1c values (both *p* for nonlinearity < 0.05; Figure 3). In the final model, threshold analysis and segmented regression revealed an inflection point at TSH = 5.53 mIU/L. When TSH < 5.53 mIU/L, each unit increase in TSH was associated with a 0.179% decrease in HbA1c. However, no significant association was observed when TSH < 5.53 mIU/L (TSH < 5.53: *β* = −0.179, 95% CI (−0.281, −0.076); TSH > 5.53: *β* = 0.042, 95% CI (−0.046, 0.130); *p* for log‐likelihood ratio = 0.005; Figure 3; Table 4).

**TABLE 4:** Threshold analysis of the effect of TSH on HbA1c.

	Crude *β* (95% CI)	Adjusted *β* (95% CI)
Fitting model by standard linear regression	−0.009 (−0.146–0.034)	−0.055 (−0.111–0)
Fitting model by two‐piecewise linear regression inflection point	4.54	5.53
TSH < inflection point	−0.267 (−0.384, −0.149)	−0.179 (−0.281,‐0.076)
TSH > inflection point	0.015 (−0.068, 0.098)	0.042 (−0.046, 0.13)
*p* for Log‐likelihood ratio	< 0.001	0.005

*Note:* Adjusted for age, gender, BMI.

Abbreviations: 95% CI: 95% confidence interval; FT3: free triiodothyronine; FT4: free tetraiodothyronine; TSH: thyroid‐stimulating hormone thyrotropin.

## 4 Discussion

### 4.1 Effect of FT3 on Blood Glucose

Our study identified a significant nonlinear association between FT3 and HbA1c, although no significant threshold effect was observed. This finding may be explained by the relatively concentrated distribution of FT3 levels in our study, as over 90% of participants exhibited values within the normal range. Within this range, thyroid hormones act synergistically with insulin to facilitate glucose disposal and utilization in peripheral tissues [24, 25]. Consequently, the influence of FT3 on HbA1c is limited [26, 27] and potentially masked by other metabolic factors. Previous studies have reported a positive correlation between BMI and FT3, as well as a significant effect of BMI on HbA1c [28, 29]. In the present study, we controlled for BMI as a confounding factor, which may have attenuated the threshold effect in the nonlinear relationship between FT3 and HbA1c.

Although no significant threshold effect was identified between FT3 and HbA1c, the observed nonlinear trend suggests a potential but modest protective effect of FT3 in glycemic regulation among patients with T2DM. Previous research has demonstrated a positive correlation between FT3 levels and pancreatic β‐cell function in pre‐diabetic populations, suggesting that FT3 may contribute to glucose homeostasis through mechanisms such as inhibiting β‐cell apoptosis and enhancing insulin gene transcription and expression [30, 31, 32]. These findings imply that FT3 should be considered a continuous regulatory factor in the glycemic management of patients with T2DM. However, the sensitivity of using FT3 alone as a clinical predictor for glycemic control is limited, necessitating an integrated interpretation in conjunction with other metabolic indicators.

However, existing evidence remains inconsistent; while Jing et al. [24] reported a negative correlation between FT3 and blood glucose, other studies have documented a positive association with fasting hyperglycemia [33]. These discrepancies underscore the necessity for large‐scale prospective cohort studies to further elucidate the dose–response relationship between FT3 and HbA1c regulation.

### 4.2 Effect of FT4 on Blood Glucose

Our study identified a significant nonlinear association between FT4 and HbA1c using restricted cubic spline modeling, with threshold analysis revealing an inflection point at 14.82 pmol/L. Specifically, when FT4 was below 14.82 pmol/L, higher FT4 values were correlated with increased HbA1c levels, whereas above this threshold, the association was not statistically significant. This finding provides a plausible explanation for the inconsistent results in previous meta‐analyses examining the FT4‐HbA1c relationship in euthyroid populations [34], as the lack of consideration for nonlinear effects could have led to the discrepant results.

From a biological perspective, FT4 may influence glucose metabolism through dual mechanisms within the low‐to‐moderate concentration range. On one hand, thyroid hormones can directly stimulate hepatic glucose production by activating key gluconeogenic enzymes, leading to elevated blood glucose levels [35, 36]. Relevant studies also indicate that individuals with lower FT4 levels exhibit higher fasting blood glucose and HbA1c levels [37], alongside an increased risk of prediabetes [36]. On the other hand, FT4 may enhance β‐adrenergic receptor sensitivity, indirectly exacerbating insulin resistance and impairing glucose utilization, thereby contributing to further elevations in HbA1c [38, 39].

However, when FT4 approaches the upper limit of the normal range, compensatory mechanisms may be activated, such as thyroid hormone receptor desensitization or upregulation of deiodinase activity, attenuating its regulatory impact on glucose metabolism [35, 40]. Thus, these homeostatic responses may explain the absence of a continued rise in HbA1c at elevated FT4 levels, suggesting that within the normal thyroid function range, FT4 fluctuations exert a limited influence on glucose homeostasis [41].

### 4.3 Effect of TSH on Blood Glucose

Threshold analysis of TSH and HbA1c in our study revealed an inflection point at 5.53 mIU/L. Specifically, TSH was negatively correlated with HbA1c when TSH was below 5.53 mIU/L, indicating a protective effect of TSH on HbA1c within this range. For patients with T2DM and normal thyroid function, maintaining TSH at relatively low levels may be more beneficial for glycemic control. However, when TSH exceeded 5.53 mIU/L, no significant correlation was observed. Previous studies on the effect of TSH on blood glucose levels have yielded inconsistent results [24, 42]. This finding challenges the traditional linear model assumption and provides a new perspective on the complex interplay between thyroid function and glucose metabolism.

The protective effects of TSH within the low‐to‐moderate concentration range reflect a compensatory mechanism in the early stages of hypothyroidism. Specifically, at lower concentrations, TSH reduces HbA1c levels by stimulating the release of free FT3 and FT4 from the thyroid gland, which could enhance insulin sensitivity and promote glucose uptake in peripheral tissues [35, 43]. However, when TSH exceeds 5.53 mIU/L, its association with HbA1c dissipates, which may indicate metabolic disturbances characteristic of the hypothyroid state. Hypothyroidism is often associated with insulin resistance and increased hepatic glucose production, both of which counteract the protective influence of TSH on glucose metabolism [44]. Furthermore, it has been suggested that the effect of TSH on HbA1c may plateau once a certain threshold is surpassed [43, 45, 46], possibly due to adaptive mechanisms such as the downregulation of insulin‐antagonistic effects or changes in thyroid hormone receptor sensitivity [47]. Thus, at higher TSH levels, the relationship between TSH and HbA1c may be obscured by these underlying metabolic dysfunctions. Similar findings have been reported in other studies; for example, the association between TSH and cardiovascular risk has also been shown to exhibit nonlinearity [48]. A large prospective study further suggested that TSH may offer metabolic protection at lower levels, but this effect diminishes or disappears beyond a certain threshold [49].

In this study, the associations of FT4 and TSH with HbA1c exhibit concentration‐dependent non‐linear characteristics, with 14.82 pmol/L and 5.53 mIU/L identified as the respective optimal concentration thresholds, providing potential biomarkers for glycemic management in the euthyroid population. Specifically, in individuals with FT4 < 14.82 pmol/L or TSH < 5.53 mIU/L, fluctuations in FT4 and TSH may significantly impact glucose metabolism, even within the normal range of thyroid function. Consequently, variations in thyroid hormones represent a non‐negligible factor in the glycemic management of patients with T2DM. In addition to glycemic control, regular monitoring of thyroid hormone levels is recommended. Timely clinical intervention should be initiated for patients exhibiting abnormal levels or high variability, particularly among high‐risk individuals with comorbid obesity or metabolic syndrome. While this study offers a novel perspective on the intricate relationship between thyroid hormones and glucose metabolism, further research is warranted to elucidate the underlying biological mechanisms and validate these clinical implications.

## 5 Limitation

Firstly, this study employed a retrospective design, precluding the ability to infer causality. Secondly, although potential confounders such as BMI, age, and sex were adjusted for, data regarding the use of insulin resistance indices, glucose‐lowering medications, or thyroid medications were not included, which may introduce residual confounding bias. Moreover, the thyroid hormone levels of the participants were predominantly within the reference range, which limits the generalizability of the findings to populations with abnormal thyroid function. Future longitudinal cohort or interventional studies, which account for thyroid antibody status and medication use and extend to subgroups with thyroid dysfunction, are recommended to further validate the generalizability and elucidate the mechanisms underlying the threshold effect.

## 6 Conclusion

This study is the first to identify nonlinear associations and threshold effects between FT3, FT4, TSH, and HbA1c using an innovative nonlinear analysis approach. Although the findings demonstrate an association rather than causation, they provide novel insights and evidence that support further investigation into the relationship between thyroid function and glucose metabolism. Additionally, they suggest a potential biomarker for the management of blood glucose levels in diabetic patients. Moving forward, we plan to conduct well‐designed prospective cohort studies or randomized controlled trials to further explore this association and establish causality.

## Author Contributions

**Mengjie Chen:** writing – original draft, formal analysis, investigation. **Yuqin Gan:** formal analysis, investigation, writing – review and editing. **Fengxiang Tian:** investigation, data curation, validation. **Yun Bao:** writing – review and editing, supervision, writing – Reviewing. **Zhonglan Chen:** conceptualization, methodology, writing – review and editing. All authors have read and approved the final manuscript.

## Funding

The authors have nothing to report.

## Ethics Statement

The study was approved by the Ethics Committee of First Affiliated Hospital of Chengdu Medical College (approval no. 2022CYFYIRB‐BA‐Dec01), and it was carried out in accordance with the Code of Ethics of the World Medical Association (Declaration of Helsinki).

## Consent

As this was a retrospective study that did not cause any harm to the patients, it was exempted by the Ethics Committee from the requirement of signing a patient informed consent form.

## Conflicts of Interest

The authors declare no conflicts of interest.

## Data Availability

The datasets generated during and/or analyzed during the current study are available from the corresponding author on reasonable request.

## References

[1] International Diabetes Federation , IDF Diabetes Atlas, 10th ed. (2021).

[2] National Centre for Geriatrics, Chinese Medical Association Diabetes Branch, Chinese Society Of Sports Science , “Guideline for Eexercise Therapy of Type 2 Diabetes Mellitus in China (20242024 Edition),” Chinese Journal of Sports Medicine 43, no. 06 (2024): 419–452, 10.3969/j.issn.1000-6710.2024.06.001.

[3] E. H. Elgazar , N. E. Esheba , S. A. Shalaby , and W. F. Mohamed , “Thyroid Dysfunction Prevalence and Relation to Glycemic Control in Patients With Type 2 Diabetes Mellitus,” Diabetes and Metabolic Syndrome: Clinical Research and Reviews 13, no. 4 (2019): 2513–2517, 10.1016/j.dsx.2019.07.020. 31405670

[4] American Diabetes Association , “Classification and Diagnosis of Diabetes: Standards of Medical Care in Diabetes‐2019,” Diabetes Care 42, no. 1 (2019): S13–S28, 10.2337/dc19-S002. 30559228

[5] K. Khunti , F. Zaccardi , A. Amod , et al., “Glycaemic Control Is Still Central in the Hierarchy of Priorities in Type 2 Diabetes Management,” Diabetologia 68, no. 1 (2025): 17–28, 10.1007/s00125-024-06254-w. 39155282 PMC11663178

[6] American Diabetes Association , “6.Glycemic Targets: Standards of Medical Care in Diabetes‐2021,” Diabetes Care 44, no. Suppl 1 (2021): S73–S84, 10.2337/dc21-S006. 33298417

[7] M. Stolar , “Glycemic Control and Complications in Type 2 Diabetes Mellitus,” American Journal of Medicine 123 (2010): S3–S11, 10.1016/j.amjmed.2009.12.004. 20206730

[8] L. Yu , Z. Li , R. Yang , et al., “Impaired Sensitivity to Thyroid Hormones Is Associated With Elevated Blood Glucose in Coronary Heart Disease,” Front Endocrinol (Lausanne) 13 (2022): 895843, 10.3389/fendo.2022.895843. 35784545 PMC9240192

[9] C. Cui , H. Sui , Z. Wang , et al., “Thyroid Hormone Sensitivity and Diabetes Onset: A Longitudinal Cross‐Lagged Cohort,” Front Endocrinol (Lausanne) 14 (2023): 1267612, 10.3389/fendo.2023.1267612. 37908753 PMC10613705

[10] Y. S. Eom , J. R. Wilson , and V. J. Bernet , “Links Between Thyroid Disorders and Glucose Homeostasis,” Diabetes and Metabolism Journal 46, no. 2 (2022): 239–256, 10.4093/dmj.2022.0013. 35385635 PMC8987680

[11] L. Jing , C. Yuqing , and C. Lei , “The Relationship Between Thyroid Function,Insulin Resistance and Islet β‐Cell Function in Women With Gestational Diabetes Mellitus,” Current Advances in Obstetrics and Gynecology (Chinese Journal) 30, no. 08 (2021): 593–596, 10.13283/j.cnki.xdfckjz.2021.08.003.

[12] H. Haiying , Y. Li , and Y. Yuan , “Relationship of Changes of Serum SF and Thyroid Function Index With Levels of Glucose and Lipid Metabolism and Obesity in T2DM Patients,” Medical Sciences Journal of South China 52, no. 06 (2024): 926–929, 10.15972/j.cnki.43-1509/r.2024.06.012.

[13] C. Ting , L. Lingjuan , and L. Hongbin , “The Correlation Between Thyroid Hormone Levels and HOMA‐IR, Glucose Metabolism Disorders in Patients With Hyperthyroidism,” Chinese Journal of Medical Innovation 21, no. 21 (2024): 180–183.

[14] B. R. Gauthier , A. Sola‐García , M. Á. Cáliz‐Molina , et al., “Thyroid Hormones in Diabetes, Cancer, and Aging,” Aging Cell 19, no. 11 (2020): e13260, 10.1111/acel.13260. 33048427 PMC7681062

[15] L. Xiaoliu and Y. Sejuan , “Changes of Blood Glucose Variability in Patients With Type 2 Diabetes Mellitus Complicated With Subclinical Hypothyroidism and Its Clinical,” Chinese and Foreign Medical Research 23, no. 02 (2025): 70–73, 10.14033/j.cnki.cfmr.2025.02.018.

[16] B. Biondi , G. J. Kahaly , and R. P. Robertson , “Thyroid Dysfunction and Diabetes Mellitus: Two Closely Associated Disorders,” Endocrine Reviews 40, no. 3 (2019): 789–824, 10.1210/er.2018-00163. 30649221 PMC6507635

[17] F. Xiaoli , T. Hong , L. Hui , X. Qingbing , and G. Xuelan , “A Study of the Correlation Between Subclinical Hyperthyroidism and Glycaemic Lipids in Patients With Type 2 Diabetes Mellitus,” Anhui Medical Journal (Chinese Journal) 45, no. 04 (2024): 478–482.

[18] N. Takamura , A. Akilzhanova , N. Hayashida , et al., “Thyroid Function Is Associated With Carotid Intima‐Media Thickness in Euthyroid Subjects,” Atherosclerosis 204, no. 2 (2009): e77–e81, 10.1016/j.atherosclerosis.2008.09.022. 18977482

[19] X. Sun , Y. Sun , W. C. Li , et al., “Association of Thyroid‐Stimulating Hormone and Cardiovascular Risk Factors,” Internal Medicine 54, no. 20 (2015): 2537–2544, 10.2169/internalmedicine.54.4514. 26466686

[20] S. I. Bukhari , G. Ali , M. Y. Memom , et al., “Prevalence and Predictors of Thyroid Dysfunction Amongst Patients With Type 2 Diabetes Mellitus in Pakistan,” Journal of Family Medicine and Primary Care 11, no. 6 (2022): 2739–2743, 10.4103/jfmpc.jfmpc_2106_21. PMC9480699 36119299

[21] B. LIiu , H.‐y. Liu , and X. Shen , “Clinical Analysis of Serum Lipid and Glucose Metabolism in Patients With Diabetes Mellitus,” Labelling Immunoassays and Clinical 24, no. 05 (2017): 550–553, 10.11748/bjmy.issn.1006-1703.2017.05.018.

[22] World Health Organization , “Definition, Diagnosis and Classification of Diabetes Mellitus and Its Complications: Report of a WHO Consultation. Part 1, Diagnosis and Classification of Diabetes Mellitus,” Diabetic Medicine: A Journal of the British Diabetic Association 15, no. 7 (1999): 539–553, 10.1002/(SICI)1096-9136(199807)15:7<539::AID-DIA668>3.0.CO;2-S. 9686693

[23] B. Hao , L. Lyu , J. Xu , et al., “The Relationship Between Triglyceride‐Glucose Index and Prospective Key Clinical Outcomes in Patients Hospitalised for Coronary Artery Disease,” Cardiovascular Diabetology 23, no. 1 (2024): 40, 10.1186/s12933-024-02132-2. 38254088 PMC10804527

[24] S. Jing , D. Xiaoying , X. Ying , et al., “Different Levels of Thyroid Hormones Between Impaired Fasting Glucose and Impaired Glucose Tolerance: Free T3 Affects the Prevalence of Impaired Fasting Glucose and Impaired Glucose Tolerance in Opposite Ways,” Clinical Endocrinology 80, no. 6 (2014): 890–898, 10.1111/cen.12384. 24330392

[25] B. K. Singh , R. A. Sinha , J. Zhou , et al., “Hepatic FOXO1 Target Genes Are co‐Regulated by Thyroid Hormone via RICTOR Protein Deacetylation and MTORC2‐AKT Protein Inhibition,” Journal of Biological Chemistry 291, no. 1 (2016): 198–214, 10.1074/jbc.M115.668673. 26453307 PMC4697156

[26] L. Chao , Z. Taolin , Y. Liu , D. Wanhong , and P. Shixi , “Changes in Serum Thyroid Hormone Levels and Clinical Significance in Middle‐Aged and Elderly Patients With Type 2 Diabetes Mellitus,” Journal of Medical Clinical Research 31, no. 2 (2014): 3, 10.3969/j.issn.1671-7171.2014.02.041.

[27] Q. Liying , H. Ling , Y. Xiaoling , et al., “The Variation of Thyroid Hormone Levels in Patients With Type 2 Diabetes Mellitus,” Chinese Journal of Diabetes 27, no. 12 (2019): 903–908, 10.3969/j.issn.1006-6187.2019.12.006.

[28] R. Xu , F. Huang , S. Zhang , Y. Lv , and Q. Liu , “Thyroid Function, Body Mass Index, and Metabolic Risk Markers in Euthyroid Adults: A Cohort Study,” BMC Endocrine Disorders 19, no. 1 (2019): 58, 10.1186/s12902-019-0383-2. 31174521 PMC6555987

[29] D. Hertroijs , A. Elissen , M. Brouwers , et al., “A Risk Score Including Body Mass Index, Glycated Haemoglobin and Triglycerides Predicts Future Glycaemic Control in People With Type 2 Diabetes,” Diabetes, Obesity & Metabolism 20, no. 3 (2018): 681–688, 10.1111/dom.13148. PMC5836941 29095564

[30] T. J. Chuang , J. D. Lin , C. Z. Wu , et al., “The Relationships Between Thyroid‐Stimulating Hormone Level and Insulin Resistance, Glucose Effectiveness, First‐ and Second‐Phase Insulin Secretion in Chinese Populations,” Medicine (Baltimore) 100, no. 19 (2021): e25707, 10.1097/MD.0000000000025707. 34106595 PMC8133064

[31] C. Aguayo‐Mazzucato , A. M. Zavacki , A. Marinelarena , et al., “Thyroid Hormone Promotes Postnatal Rat Pancreatic β‐Cell Development and Glucose‐Responsive Insulin Secretion Through MAFA,” Diabetes 62, no. 5 (2013): 1569–1580, 10.2337/db12-0849. 23305647 PMC3636623

[32] T. Oda , H. Taneichi , K. Takahashi , et al., “Positive Association of Free Triiodothyronine With Pancreatic β‐Cell Function in People With Prediabetes,” Diabetic Medicine 32, no. 2 (2015): 213–219, 10.1111/dme.12589. 25255697

[33] G. L. Roef , E. R. Rietzschel , C. M. Van Daele , et al., “Triiodothyronine and Free Thyroxine Levels Are Differentially Associated With Metabolic Profile and Adiposity‐Related Cardiovascular Risk Markers in Euthyroid Middle‐Aged Subjects,” Thyroid 24, no. 2 (2014): 223–231, 10.1089/thy.2013.0314. 24032604 PMC3926145

[34] R. Mullur , Y. Y. Liu , and G. A. Brent , “Thyroid Hormone Regulation of Metabolism,” Physiological Reviews 94, no. 2 (2014): 355–382, 10.1152/physrev.00030.2013. 24692351 PMC4044302

[35] S. W. Kim , J. H. Jeon , J. S. Moon , et al., “Low‐Normal Free Thyroxine Levels in Euthyroid Male Are Associated With Prediabetes,” Diabetes and Metabolism Journal 43, no. 5 (2019): 718–726, 10.4093/dmj.2018.0222. 30968614 PMC6834837

[36] J. J. Garduño‐Garcia , U. Alvirde‐Garcia , G. López‐Carrasco , et al., “TSH and Free Thyroxine Concentrations Are Associated With Differing Metabolic Markers in Euthyroid Subjects,” European Journal of Endocrinology 163, no. 2 (2010): 273–278, 10.1530/EJE-10-0312. 20516204

[37] L. Raets , C. Minschart , A. Van den Bruel , et al., “Higher Thyroid fT3‐to‐fT4 Ratio Is Associated With Gestational Diabetes Mellitus and Adverse Pregnancy Outcomes,” Journal of Clinical Medicine 11, no. 17 (2022): 5016, 10.3390/jcm11175016. 36078946 PMC9457218

[38] X. Zhao , J. Sun , S. Xin , and X. Zhang , “Predictive Effects of FT3/FT4 on Diabetic Kidney Disease: An Exploratory Study on Hospitalized Euthyroid Patients With T2DM in China,” Biomedicine 11, no. 8 (2023): 2211, 10.3390/biomedicines11082211. PMC10452238 37626708

[39] G. A. Brent , “Mechanisms of Thyroid Hormone Action,” Journal of Clinical Investigation 122, no. 9 (2012): 3035–3043, 10.1172/JCI60047. 22945636 PMC3433956

[40] A. Roos , S. J. Bakker , T. P. Links , R. O. Gans , and B. H. Wolffenbuttel , “Thyroid Function Is Associated With Components of the Metabolic Syndrome in Euthyroid Subjects,” Journal of Clinical Endocrinology and Metabolism 92, no. 2 (2007): 491–496, 10.1210/jc.2006-1718. 17090642

[41] D. O. Roa , A. C. Van der Burgh , T. Ittermann , et al., “Thyroid Function and the Risk of Prediabetes and Type 2 Diabetes,” Journal of Clinical Endocrinology and Metabolism 107, no. 6 (2022): 1789–1798, 10.1210/clinem/dgac006. 35137143 PMC9315162

[42] H. Chen , J. Wu , and R. Lyu , “Expressions of Glycemic Parameters, Lipid Profile, and Thyroid Hormone in Patients With Type 2 Diabetes Mellitus and Their Correlation,” Immunity, Inflammation and Disease 12, no. 7 (2024): e1282, 10.1002/iid3.1282. 38967365 PMC11225078

[43] M. M. Bos , N. A. van Vliet , S. P. Mooijaart , R. Noordam , and D. van Heemst , “Genetically Determined Higher TSH Is Associated With a Lower Risk of Diabetes Mellitus in Individuals With Low BMI,” Journal of Clinical Endocrinology and Metabolism 106, no. 7 (2021): e2502–e2511, 10.1210/clinem/dgab277. 33901276 PMC8208661

[44] V. L. Langén , T. J. Niiranen , P. Puukka , et al., “Thyroid‐Stimulating Hormone and Risk of Sudden Cardiac Death, Total Mortality and Cardiovascular Morbidity,” Clinical Endocrinology 88, no. 1 (2018): 105–113, 10.1111/cen.13472. 28862752

[45] C. G. Ma and Y. S. Shim , “Association of Thyroid‐Stimulating Hormone and Thyroid Hormones With Cardiometabolic Risk Factors in Euthyroid Children and Adolescents Aged 10‐18 Years: A Population‐Based Study,” Scientific Reports 9, no. 1 (2019): 15476, 10.1038/s41598-019-51963-7. 31664103 PMC6820776

[46] E. Ramouzi , K. Sveroni , M. Manou , et al., “The Impact of Thyroid Hormones on Cardiometabolic Risk in Children and Adolescents With Obesity, Overweight and Normal Body Mass Index (BMI): A One‐Year Intervention Study,” Nutrients 16, no. 16 (2024): 2650, 10.3390/nu16162650. 39203787 PMC11357135

[47] L. Chaker , S. Ligthart , T. I. Korevaar , et al., “Thyroid Function and Risk of Type 2 Diabetes: A Population‐Based Prospective Cohort Study,” BMC Medicine 14, no. 1 (2016): 150, 10.1186/s12916-016-0693-4. 27686165 PMC5043536

[48] N. Takamura , N. Hayashida , and T. Maeda , “Risk of Coronary Heart Disease and Mortality for Adults With Subclinical Hypothyroidism,” Journal of the American Medical Association 304, no. 22 (2010): 2481–2482; author reply 2482, 10.1001/jama.2010.1787. 21139106

[49] Q. Hou , H. Zou , S. Zhang , et al., “Association of Maternal TSH and Neonatal Metabolism: A Large Prospective Cohort Study in China,” Front Endocrinol (Lausanne) 13 (2022): 1052836, 10.3389/fendo.2022.1052836. 36531456 PMC9753981

